# Randomized controlled trial of individualized arousal-biofeedback for children and adolescents with disruptive behavior disorders (DBD)

**DOI:** 10.1007/s00787-023-02368-5

**Published:** 2024-02-08

**Authors:** Pascal-M. Aggensteiner, Boris Böttinger, Sarah Baumeister, Sarah Hohmann, Stefan Heintz, Anna Kaiser, Alexander Häge, Julia Werhahn, Christoph Hofstetter, Susanne Walitza, Barbara Franke, Jan Buitelaar, Tobias Banaschewski, Daniel Brandeis, Nathalie E. Holz

**Affiliations:** 1 Department of Child and Adolescent Psychiatry and Psychotherapy, Central Institute of Mental Health, Medical Faculty, Mannheim/Heidelberg University, Mannheim, Germany; 2 Department of Child and Adolescent Psychiatry and Psychotherapy and Psychosomatics, University Medical Center Hamburg-Eppendorf, Hamburg, Germany; 3 Department of Child and Adolescent Psychiatry and Psychotherapy, University Hospital of Psychiatry Zurich, University of Zurich, Zurich, Switzerland; 4 Neuroscience Center Zurich, University and ETH Zurich, Zurich, Switzerland; 5 Donders Institute for Brain, Cognition and Behavior, Radboud University Nijmegen, Nijmegen, The Netherlands; 6 Department for Cognitive Neuroscience, Radboud University Medical Center Nijmegen, Nijmegen, The Netherlands; 7 Institute of Medical Psychology and Medical Sociology, University Medical Center Schleswig Holstein, Kiel University, Kiel, Germany

**Keywords:** Skin conductance, Biofeedback, Randomized controlled trial, Subtypes, Conduct disorder, Oppositional defiant disorder, Arousal, Personalized treatment, Self-regulation, Disruptive behavior disorders, Aggression

## Abstract

Disruptive behavior disorders [including conduct disorder (CD) and oppositional defiant disorder (ODD)] are common childhood and adolescent psychiatric conditions often linked to altered arousal. The recommended first-line treatment is multi-modal therapy and includes psychosocial and behavioral interventions. Their modest effect sizes along with clinically and biologically heterogeneous phenotypes emphasize the need for innovative personalized treatment targeting impaired functions such as arousal dysregulation. A total of 37 children aged 8–14 years diagnosed with ODD/CD were randomized to 20 sessions of individualized arousal biofeedback using skin conductance levels (SCL-BF) or active treatment as usual (TAU) including psychoeducation and cognitive–behavioral elements. The primary outcome was the change in parents´ ratings of aggressive behavior measured by the Modified Overt Aggression Scale. Secondary outcome measures were subscales from the Child Behavior Checklist, the Inventory of Callous-Unemotional traits, and the Reactive-Proactive Aggression Questionnaire. The SCL-BF treatment was neither superior nor inferior to the active TAU. Both groups showed reduced aggression after treatment with small effects for the primary outcome and large effects for some secondary outcomes. Importantly, successful learning of SCL self-regulation was related to reduced aggression at post-assessment. Individualized SCL-BF was not inferior to active TAU for any treatment outcome with improvements in aggression. Further, participants were on average able to self-regulate their SCL, and those who best learned self-regulation showed the highest clinical improvement, pointing to specificity of SCL-BF regulation for improving aggression. Further studies with larger samples and improved methods, for example by developing BF for mobile use in ecologically more valid settings are warranted.

**Supplementary Information:**

The online version contains supplementary material available at 10.1007/s00787-023-02368-5.

## Introduction

Oppositional defiant disorder (ODD) and conduct disorder (CD) are disruptive behavior disorders with a high prevalence ranging from 2 to 4% [19] in youth and are among the leading causes of referral to mental health services in children and youths [7]. Aggression-related problems are treated with modest cost–benefit effects. Stimulant (i.e., methylphenidate), and neuroleptic (i.e., Risperidone) treatments showed significant effects on comorbid aggression in attention-deficit/hyperactivity disorder (ADHD) patients. However, pharmacotherapy for aggression is limited by the low number of high-quality studies (RCTs) and reports of serious adverse effects [18]. Likewise, nonpharmacological psychosocial interventions show only small clinical effects [4]. The limitations of current behavioral and pharmacological treatments of pediatric aggression (Scotto [23] emphasize the need for innovative personalized treatment.

Considerable evidence suggests that arousal dysregulation is a robust psychophysiological correlate of aggression [16, 21, 27]. Initial studies suggested that electrodermal activity measured by skin conductance level (SCL) is generally lower in children, adolescents, and adults with DBDs compared to matched controls, indicating hypoarousal as shown in a meta-analysis by Lorber et al. [16], which comprised 32 studies and included a total sample size (N) of 1453. In addition, evidence suggests heterogeneity with respect to arousal dysregulation profiles depending on different subtypes of DBD. As such, hypoarousal or reduced SCL has been related to callous-unemotional traits and psychopathy [13, 16, 31], and increased SCL to reactive and impulsive aggression subtype in a typically developing sample of 272 participants [14]. However, in our recent study, involving 48 patients, we could not replicate those findings [3].

Personalized arousal-targeting interventions using biofeedback (BF) might thus be particularly promising treatment approaches. BF is characterized by training the self-regulation of a (partly) covert physiological state or response, such as SCL or heart rate, which have been associated with the person’s behavior problems. In turn, this physiological state is fed back to the person enabling a learning process to control these responses. A crucial factor for efficacy might be whether the success in learning self-regulation is successful. Indeed, clinical improvement has been particularly observed in individuals with psychiatric [24, 25] or neurological disorders [17] who were able to learn arousal self-regulation with SCL biofeedback.

Therefore, we designed a personalized SCL arousal-biofeedback training to reduce aggression in CD/ODD given the evidence for arousal dysregulation in these conditions on a behavioral level as a function of aggression subtypes with the aim of evaluating its efficacy and the relation to SCL self-regulation learning.

## Methods

### Study design and participants

Participants in the current study were recruited as a part of the EU-Aggressotype project, conducted by two different sites (Mannheim and Zurich) during 2016 and 2018. Ethical approval for the study was obtained for both sites separately from local ethics committees. Written informed consent was given by the participants and their parents or legal representatives. This multicentre, randomized-controlled, parallel group, open-label trial was registered under ClinicalTrials.gov Identifier: NCT02485587. Participants had to meet the diagnosis of ODD and/or CD based on the structured diagnostic interviews with child and parents using the Kiddie Schedule for Affective Disorders and Schizophrenia (K-SADS) [15] according to DSM-5 criteria, or scored above the clinical cut-off for aggressive behavior and/or rule-breaking behavior as measured with the Child Behavior Checklist completed by parents (CBCL, [1]. Exclusion criteria for all participants were an IQ < 80 measured from four subtests (vocabulary, similarities, block design, and picture completion/matrix reasoning) of the Wechsler Intelligence Scale for Children-IV [30] and a primary DSM-5 diagnosis of psychosis, bipolar disorder, major depression, and/or an anxiety disorder. Medication had to be stable during the treatment and at least 2 weeks prior to the inclusion. Participants were randomly allocated (1:1 ratio) to one of two treatment arms, either the experimental group receiving the arousal biofeedback training or to the active comparator group with TAU (treatment as usual) (for details see supplement).

**Fig. 1:**
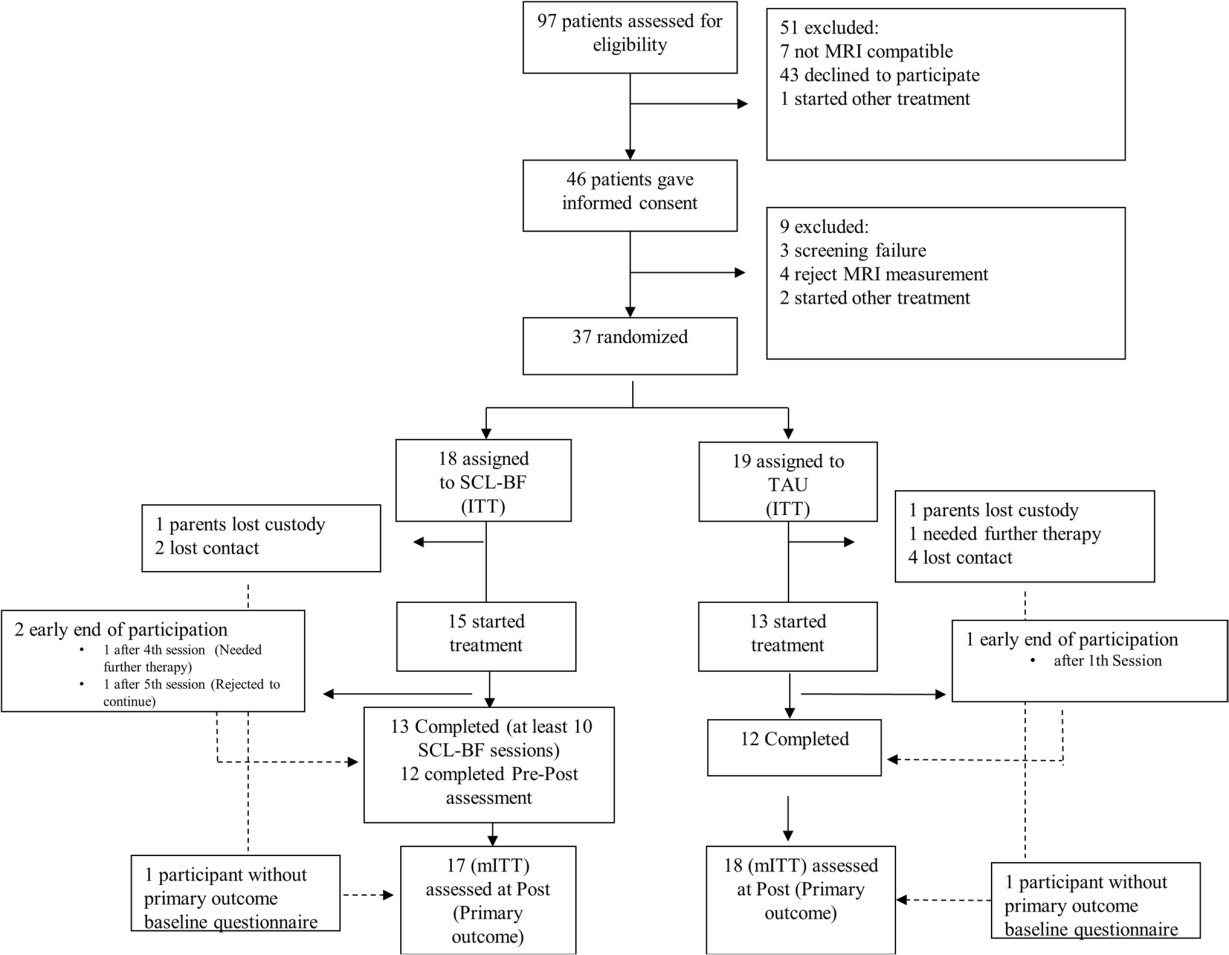
A total of 97 patients were contacted between June 2015 and April 2019 for screening, and 46 patients signed the informed consent and took part in the first assessment phase in which 9 did not meet the inclusion criteria. Finally, 37 (100%) were randomly allocated to one of the two treatment groups and 28 (75%) participants started the treatments. The ITT (intention to treat) population consisted of 18 (49%) participants in the SCL-BF and 19 (51%) in the TAU group. A total of 25 participants completed BF (*n* = 13) and TAU (*n* = 12) treatment. At post assessment 17 SCP-BF and 18 TAU participants could be analyzed using the mITT (modified intention to treat) sample. Missing values were replaced using the conservative baseline observed carried forward method (BOCF)

A total of 97 patients were contacted between June 2015 and April 2019 for screening, and 46 patients signed the informed consent from whom 37 meet the inclusion criteria. Finally, 37 (100%) were randomly allocated to one of the two treatment groups and 28 (75%) participants actually started the treatments. The CONSORT flow diagram is shown in Fig. 1. The ITT (intention-to-treat) population consisted of 18 (49%) participants in the SCL-BF and 19 (51%) in the TAU group. A total of 24 participants completed all assessments (SCL-BF = 12; TAU = 12), representing the completers sample and 13 participants participated in at least 10 SCL-BF sessions, which were used to analyze SCL-BF learning. Although the study design originally included a 6-month follow-up assessment, this report will present data based only on pre- and post-assessment, as more than 65% of the participants in each group did not participate in the 6-month follow-up (for details, see supplement).

### Skin conductance arousal biofeedback (SCL-BF)

Prior to the first biofeedback session, a subtyping assessment was performed [3]. This arousal subtyping consisted of two 3-min resting-state assessments. Each participant was classified into a hypoarousal or hyperarousal subgroup, based on the previous evidence of an SCL cut-off of 14 µS in 26 DBD patients compared to 26 matched controls [27]. Thus, participants who had high baseline SCL (> 14 µS) were to primarily down-regulate the SCL (40% up, 60% down) and participants with lower levels (< 14 µS) to primarily up-regulate the SCL (60% up, 40% down). We decided to train in both directions to promote differential self-regulation ability of the SCL. Eleven out of 13 BF participants had to mainly up-regulate their SCL (60/40%) levels.

**Fig. 2:**
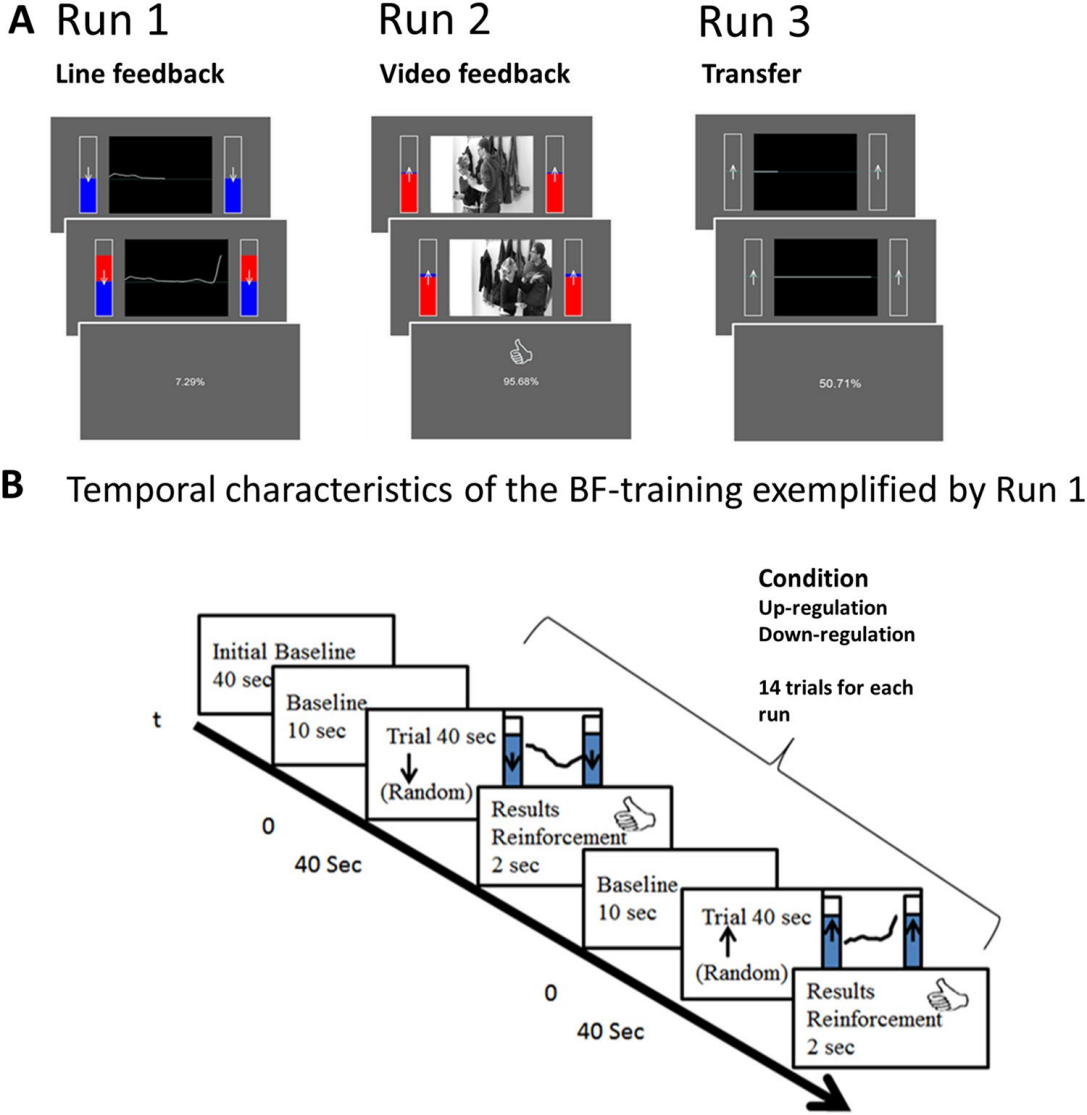
**A** Real display of each feedback run. The three runs are only different in their display mode: Run 1 is with real-time SCL feedback (line and thermometers); Run 2 is with an emotional video clip and feedback thermometers; Run 3 is without any feedback. Just the time sequence was shown as a horizontal line. **B** Time flow of one run. Each run lasted about 12 min, and a whole session approx. 1 h. Initial Baseline of 40 s was assessed. Prior to each trial we assessed a baseline (10 s), which then served as the threshold to up- or down-regulate SCL. Each regulation trial lasted 40 s. After each trial, performance accuracy was shown. Reinforcement criteria were set up to 60%

The individualized SCL-BF consisted of 20 training sessions within 20 weeks (Fig. 2). Each session had three different training runs (distinct types of feedback), and each run contained 14 trials each from two conditions (down- and up-regulate their SCL). Prior to each trial, we assessed a baseline (10 s), which then served as the threshold to up- or down-regulate SCL. Each regulation trial lasted 40 s. The first run consisted of direct feedback, and the second run included a more realistic environment for SCL regulation through affective video sequences. The third run was a transfer run, in which the participants were instructed to up- or down-regulate without any visual presentation of feedback (neither their skin conductance line nor emotional video clips) to facilitate the transfer of the learned skills into daily life. Participants were reinforced with a thumb up, if they managed to stay above or below the baseline during equal or more than 60% of the trial duration. The instruction given to the participants can be found in the supplementary material. Furthermore, we implemented a token system, in which the participants could collect points at each training session for successful performance (where 1 point equaled three collected thumbs-ups, with a theoretical maximum of 80 points achievable in total), as well as for treatment compliance (1 point awarded per day for regular participation and an additional 1 point per day for compliance during training). These accumulated points were further rewarded with a voucher worth €10, allowing participants to choose their preferred reward for every unit of 40 collected points. Additionally, we systematically addressed temper tantrums by setting clear session expectations, utilizing the token system to encourage cooperation, and ensuring safety through a passive approach and a well-equipped lab during uncooperative incidents.

SCL was recorded using a CE-certified 22-channel TheraPrax® Q-EEG-System (NeuroConn GmbH, Illmenau, Germany). Ag–AgCl electrodes on the third and fourth finger of the non-dominant hand were centered at the volar surface of the distal phalanges and filled with an electrolyte gel (TD-246 Electrode Paste, Discount Disposables, Vermont, USA). The room temperature was kept at 22–24° to prevent influence of temperature on SCL, and each training session lasted about one hour.

### Treatment as usual

The active control group TAU consisted of 6 sessions within 20 weeks of individual psychoeducation and counseling with cognitive–behavioral elements offered and matched in the recruiting institutions. After a first session with caregivers and patient together, four sessions with the patient alone took place. The final sixth session was a further family therapy session. Cognitive–behavioral treatment consisted of selected elements of standardized manuals for the training of social competencies and aggression (Soziales Kompetenztraining (SKT), Anti-Aggressivitäts-Training (AAT; 29) and Assertiveness-Training-Program (ATP; Pfingsten 2000), which were individually combined to meet the personal needs of each participant. Each session lasted about 1 h.

### Primary outcome

The primary outcome measure was the modified version of the Overt Aggression Scale (MOAS) [26] in which parents or caregivers had to rate the aggressive behavior of the participants retrospectively for the last seven days. The MOAS, like the OAS [32] on which it is based, is designed to measure four types of aggression (verbal, against objects, against self, and against others) by severity and frequency, with each type having a rating of zero when the type of aggression was absent, and four levels of severity (for details, see supplement). The MOAS questionnaire shows a good validity and test–retest reliability (0.75) [6]

### Secondary outcomes

The secondary outcome measures consisted of the following questionnaires: the Child Behavior checklist (CBCL, [1] with its subdomains rule-breaking behavior, aggression subscale, ADHD and anxiety symptoms, the Inventory of Callous-Unemotional Traits (ICU) [10] rated by parents, and the self-reported Reactive and Proactive Questionnaire (RPQ) [22]. All questionnaires are widely used with good-to-excellent validity and test–retest reliabilities ranging from 0.95 to 1.00 for the CBCL [1], 0.70 to 0.81 for the ICU [8] and the RPQ demonstrated test–retest reliability range from 0.84 to 0.86 [22].

## Statistical analysis

### Demographics

Group differences in demographic variables were analyzed using analysis of variance (ANOVA) or Chi-square tests, when appropriate. Additionally, effect sizes (ES) were reported using the mean difference and the pooled weighted standard deviation corrected for reduced sample sizes [12]. ES can range between small (< 0.5), medium (0.5–0.8), and large (> 0.8).

### Clinical outcome

The treatment effect on the primary outcome (MOAS) and the secondary outcomes was tested by a repeated measures (RM)-ANOVA with a between factor of group and a within factor of time (Pre–Post-treatment). We first analyzed the data using the mITT (modified intention-to-treat) population. Missing values were replaced using the conservative baseline observed carried forward method (BOCF). Additionally, the same analysis was restricted to participants who had completed all assessments, and sensitivity analyses were performed including age and IQ as covariates. All statistical analysis except for the treatment group by time ANOVA of the pre-registered primary outcome (MOAS) are considered exploratory. Clinical data were analyzed using SPSS version 25. Based on a posterior power analysis conducted using R Studio version 4.12 and the WebPower package, our study determined that with a sample size of 35 (modified intention-to-treat population, mITT), a medium minimum detectable between-groups effect size of *f* = 0.48 was revealed with an 80% power.

### Biofeedback learning

Linear mixed models were used to test whether the SCL-BF participants were able to learn and improve self-regulation of their SCL. Dependent variable included session performance (% of correct regulation) over the 20 biofeedback sessions. The linear mixed model included fixed effects for session, run and condition (up/down-regulation). Run and condition were set as factors. A random intercept of participants and a random slope for session were included in the model. The model further included interaction terms between session and condition and session and run. Significant interactions were followed up by a simple slope analysis. Further, individual learning for each participant was determined using the slope over the SCL-BF sessions and correlated with clinical outcome using Spearman rank correlation. Two participants in the SCL-BF group had less than ten training sessions and were excluded from the analysis. Additionally, we correlated the number of attended sessions with the clinical outcome for all participants. Biofeedback learning were analyzed with R studio (lme4 and interaction package).

## Results

From the 37 randomized participants, 17 had an ODD diagnosis (SCL-BF = 10; TAU = 7), 9 ODD/CD (SCL-BF = 4; TAU = 5), and 3 CD (SCL-BF = 2; TAU = 1) alone and 8 (SCL-BF = 2; TAU = 6), presented a T-score > 70 on the aggressive behavior and/or rule-breaking behavior subscale. Furthermore, 6 participants also had comorbid ADHD (SCL-BF = 3; TAU = 3). Baseline characteristics did not differ between groups, except for higher RPQ scores in the BF group. Details are depicted in Table 1.

**Table 1:** Baseline characteristics ITT population

	SCL-BF *n* = 18		TAU *n* = 19		*p* value
	Mean (SD)	Range	Mean (SD)	Range	
Age (years)	11.2 (2.09)	8.02–14.36	11.1 (1.88)	8.00–14.09	0.89
Male	17 (94%)		18(94%)		1.0
IQ^a^	103 (9.72)	83–115	106 (9.67)	89–118	0.46
Medication prior to study	7(39%)		9(47%)		0.74
MOAS^a^	10.4 (8.78)	0–28	8.4(7.1)	0–24	0.46
RPQ^b^	18.2 (10.4)	1.0–43	11.8(5.0)	4.0–20	**0.04**
ICU^a^	33.2(9.8)	15–51	33.11(8.5)	21–47	0.96
CBCL *t* value^a^					
Global	67.7 (7.09)	53–76	67.8 (5.65)	54–77	0.97
Externalizing problems	71.5 (7.89)	57–80	70.3 (5.66)	59–82	0.58
ODD	68.6 (7.29)	57–80	68.9 (4.34)	58–75	0.84
CD	72.3 (11.3)	51–89	70.1 (7.75)	53–86	0.48
Internalizing problems	62.9 (7.81)	45–71	61.8 (7.81)	47–74	0.68

IQ estimated from a subset of the Wechsler Intelligence Scale for Children III. MOAS, Modified Overt Aggression Scale; CBCL, Child Behavior Checklist; ICU, Inventory of Callous-Unemotional Traits; RPQ, Reactive–proactive Questionnaire

^a^*n* = 17/18, ^b^*n* = 16/15; *X*^2^ Chi-square. Participants who where medicated predominantly received either stimulants only (SCL-BF = 4; TAU = 7), a combination with stimulants and antipsychotics (SCL-BF = 2) or a combination with non-stimulants (SCL-BF = 1; TAU = 2)

### Primary outcome

Two participants were excluded due to missing baseline data. In total, 35 mITT participants were analyzed. RM-ANOVA of the MOAS questionnaire showed a significant effect of time [F(1,33) = 6.57, *p* = 0.015] with a small-effect size (ES = 0.27, [CI 95% = 0.41–0.498]), and irrespective of group (*p* = 0.208). This result did not change when only completers were analyzed (ES = 0.38, [CI 95% = 0.046–0.715], *p* = 0.024). Sensitivity analyses, including IQ and age as covariates, yielded the same results as the main analyses but revealed also that participants with lower IQ (*p* = 0.008) and younger age (*p* = 0.049) improved more after treatment, irrespective of group. Additionally, correlation analysis revealed that IQ (*r* = − 0.428, *p* = 0.011), but not age (*r* =− 0.149, *p* = 0.25) correlated significantly with clinical change. Exploratory within-group analysis between pre- and post-assessments were significant for the SCL-BF group only (ES = 0.36, [CI 95% = 0.036–0.689], *p* = 0.020). For details, Table 2.

**Table 2:** Within effect sizes for both groups

	Pre		Post		Group differences	Within groups ES (Hedges)	CI 95%	Pre–post (*t* test) p value
	Mean	SD	Mean	SD				
MOAS^a^								
BF	10.41	8.78	7.23	8.22	ns	0.36	0.036–0.689	0.020
TAU	8.39	7.18	7.33	6.33		0.15	− 0.188–0.489	0.355
Externalyzing symptoms^b^								
BF	29.33	13.87	15.42	10.71	ns	1.08	0.158–2.001	0.010
TAU	25.83	8.17	21.00	10.85		0.48	− 0.238–1.198	0.149
Oppositional defiant symptoms^b^								
BF	7.08	1.88	5.08	2.47	ns	0.88	− 0.103–1.866	0.053
TAU	6.75	1.29	5.50	2.24		0.37	− 0.218–1.530	0.105
Conduct disorder^b^								
BF	11.42	7.44	5.67	5.35	ns	0.84	0.048–1.640	0.021
TAU	10.58	4.54	9.08	4.78		0.31	− 0.332–0.953	0.298
Inventory of Callous− unemotional traits^b^								
BF	31.08	7.14	28.50	11.98	ns	0.22	− 0.262–0.708	0.322
TAU	32.00	6.97	33.58	7.23		− 0.21	− 0.845–0.414	0.462
RPQ total^c^								
BF	16.73	6.32	14.27	10.38	ns	0.26	− 0.396–0.916	0.388
TAU	12.60	5.58	12.10	10.60		0.04	− 0.400–0.488	0.828
RPQ reactive^c^								
BF	12.82	4.00	10.45	5.42	ns	0.47	− 0.375–1.324	0.224
TAU	10.10	4.63	8.60	6.42		0.24	− 0.256–0.733	0.288
RPQ proactive^c^								
BF	3.91	3.67	3.50	3.71	ns	0.14	− 0.472–0.501	0.949
TAU	3.50	4.53	4.40	5.02		− 0.17	− 0.510–0.157	0.244

BF: Biofeedback; TAU: Treatment as usual; ns: not significant; RPQ: Reactive and proactive questionnaire

^a^*N* = 17/18 (mITT), ^b^*N* = 12/12, ^c^*N* = 11/10

### Secondary outcomes

For the CBCL, we found lower externalizing symptoms after treatment in both groups [*F*(1,22) = 11.699, *p* = 0.002] with a large-effect size (ES = 0.83, [CI 95% = 0.251–1.41]). Regarding the subdomains of the CBCL, which reflect the core domains of aggression-related symptoms, medium-to-large improvements were obtained for the ODD subscale [*F*(1,22) = 7.822, *p* = 0.011, ES = 0.81, CI 95% = 0.168–1.444] and for the CD subscale [*F*(1,22) = 8.151, *p* = 0.009, ES = 0.63, CI 95% = 0.118–1.138]. However, again, no significant group differences were found. In an exploratory within-group analysis, pre–post-differences showed medium-to-large ES and were only significant in the BF group, and not in the TAU group.

No significant changes were found in CU traits and RPQ total score and its subscales (all *p* > 0.152). All treatment and time effects are depicted in Table 2.

### Biofeedback learning and clinical outcome

Mean effect of session was not significant (*p* = 0.199). However, a significant session x condition interaction emerged (*p* = 0.046), which revealed an increase in performance over time for the up-regulation condition across runs. Exploratory post hoc between-session comparisons revealed significant improvement between the first session and the eleventh (*p* = 0.0107), thirteenth (*p* = 0.0168), fourteenth (*p* = 0.0225), and sixteenth (*p* = 0.0442) session for the up-regulation condition. In addition, self-regulation for the up-regulation condition had lower mean percent of correct regulation, indicating that it was more difficult to carry out (up- vs down-regulation, *p* < 0.001). With regard to the different runs, the transfer run proved to be most difficult (*p* = 0.003).

**Fig. 3:**
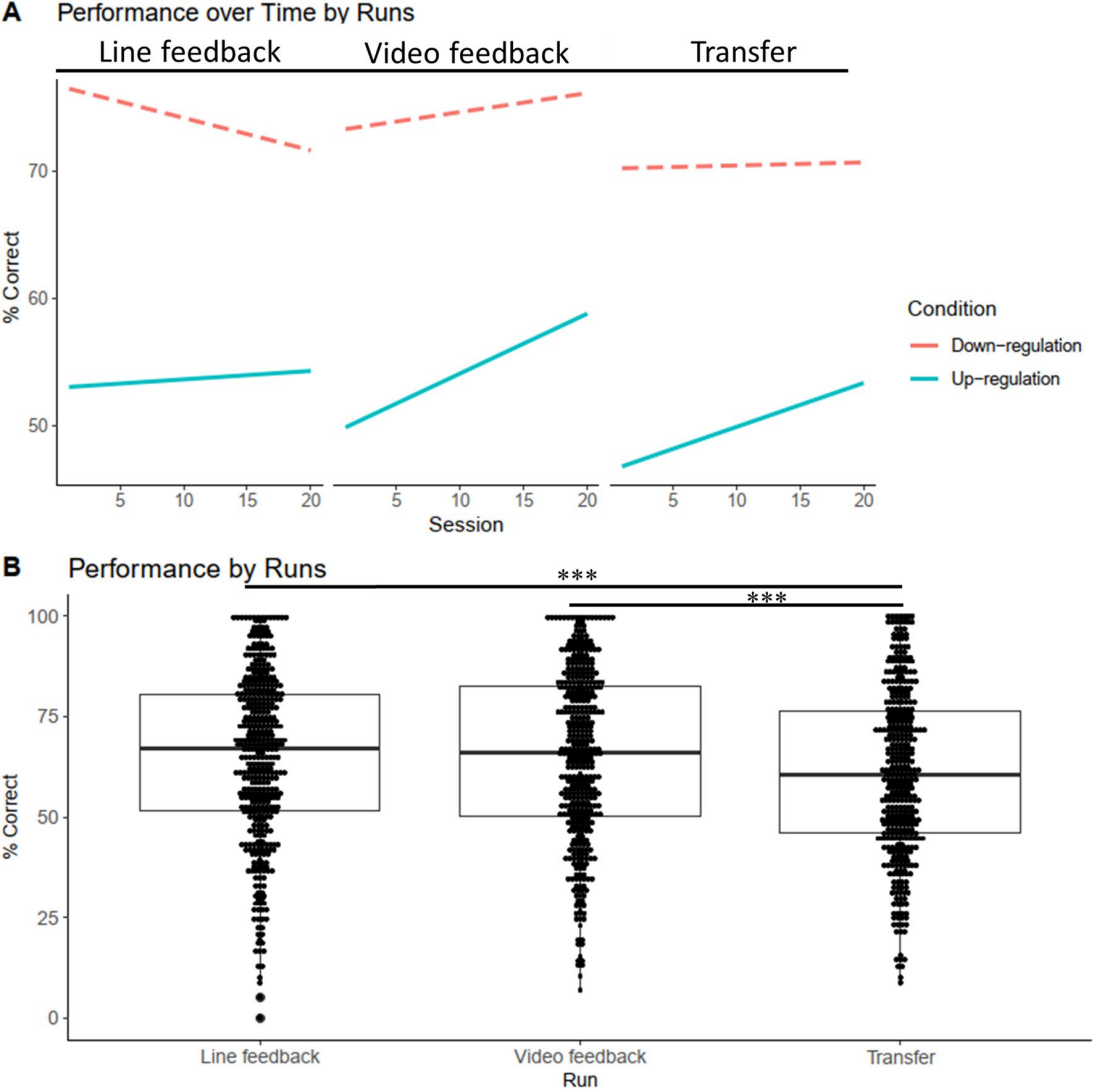
SCL-BF Performance. **A** SCL-BF performance across sessions and runs. **B** Mean performance for each run. ***p < 0.001

Additionally, an interaction between session and run emerged, revealing that improvement over time was higher in the video run (*p* = 0.046). For details, see Fig. 3 and Table 3. For individual performance over time and a secondary analysis using the offline preprocessed skin-conductance data, which revealed a session x condition interaction at a trend level only (*p* = 0.051). Furthermore, we assessed if medication affected the SCL-BF learning but found no impact on the main model. See supplementary material for more details.

**Table 3:** Mixed model for Biofeedback performance over

Predictors	Estimates	CI	Statistic	*p*
(Intercept)	76.72	71.54–81.90	29.08	< 0.001
Session	**− **0.25	**− **0.64–0.13	**− **1.29	0.199
Run [2] vs Run [1]j	− 3.57	**− **7.87–0.73	**− **1.63	0.787
Run [3] vs Run [1]	− 6.51	**− **10.83–**− **2.19	**− **2.96	**0.001**
Cond [Up] vs Cond [Down]	− 23.70	**− **27.23–**− **20.18	**− **13.20	** < 0.001**
Session * Cond [Up]	0.32	0.01–0.64	2.00	**0.046**
Session * Run [2]	0.40	0.02–0.79	2.06	**0.040**
Session * Run [3]	0.28	− 0.11–0.66	1.42	0.157

In bold significant results

**Fig. 4:**
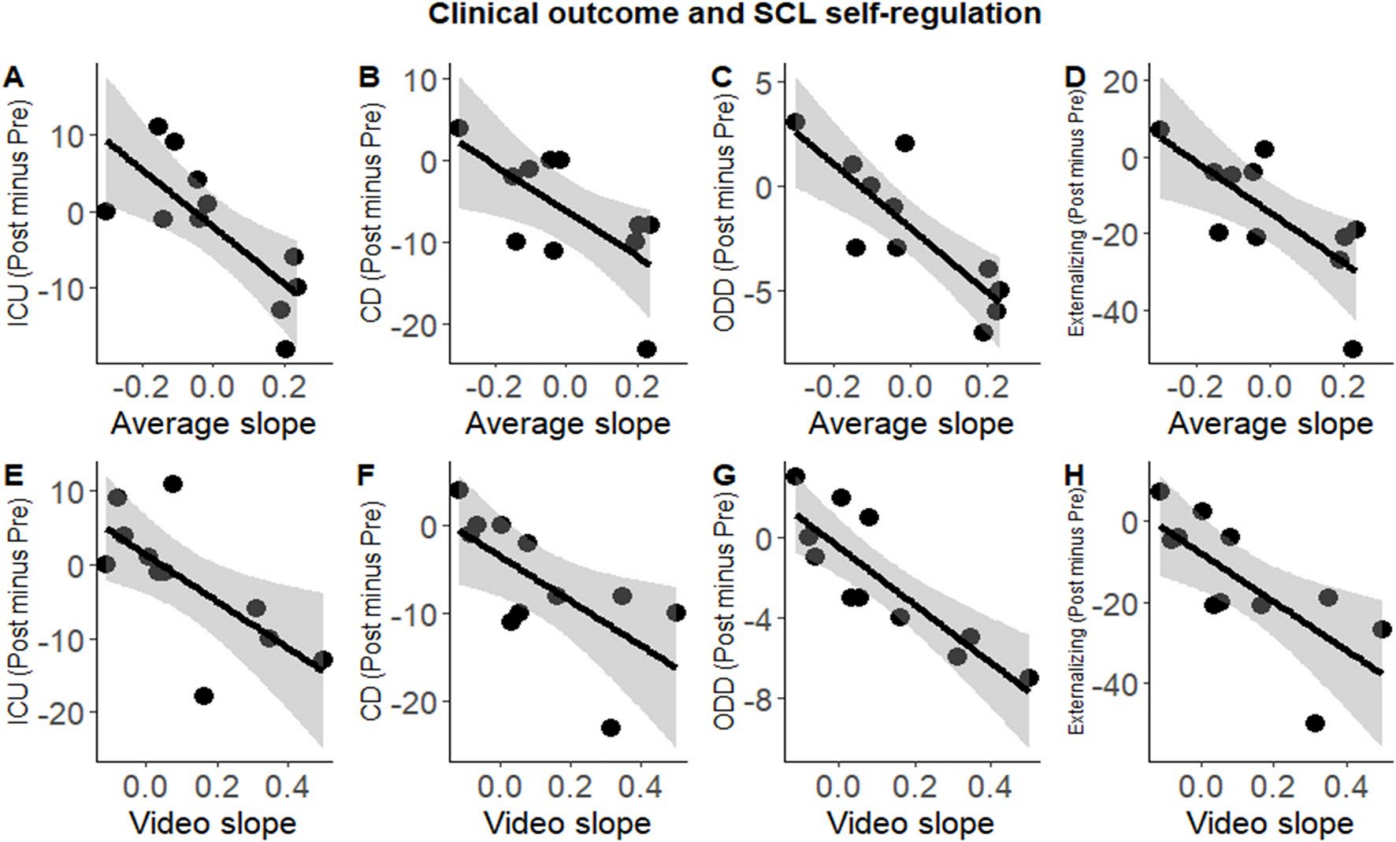
Clinical outcome and SCL self-regulation improvement. Negative signs indicates more clinical improvement (Post–Pre), and positive slope better SCL performance. **A** ICU: Inventory of callous-unemotional traits. **B** CD: Conduct disorder. **C** ODD: Oppositional defiant disorder. **D** Externalizing symptoms (CBCL)

**Fig. 5:**
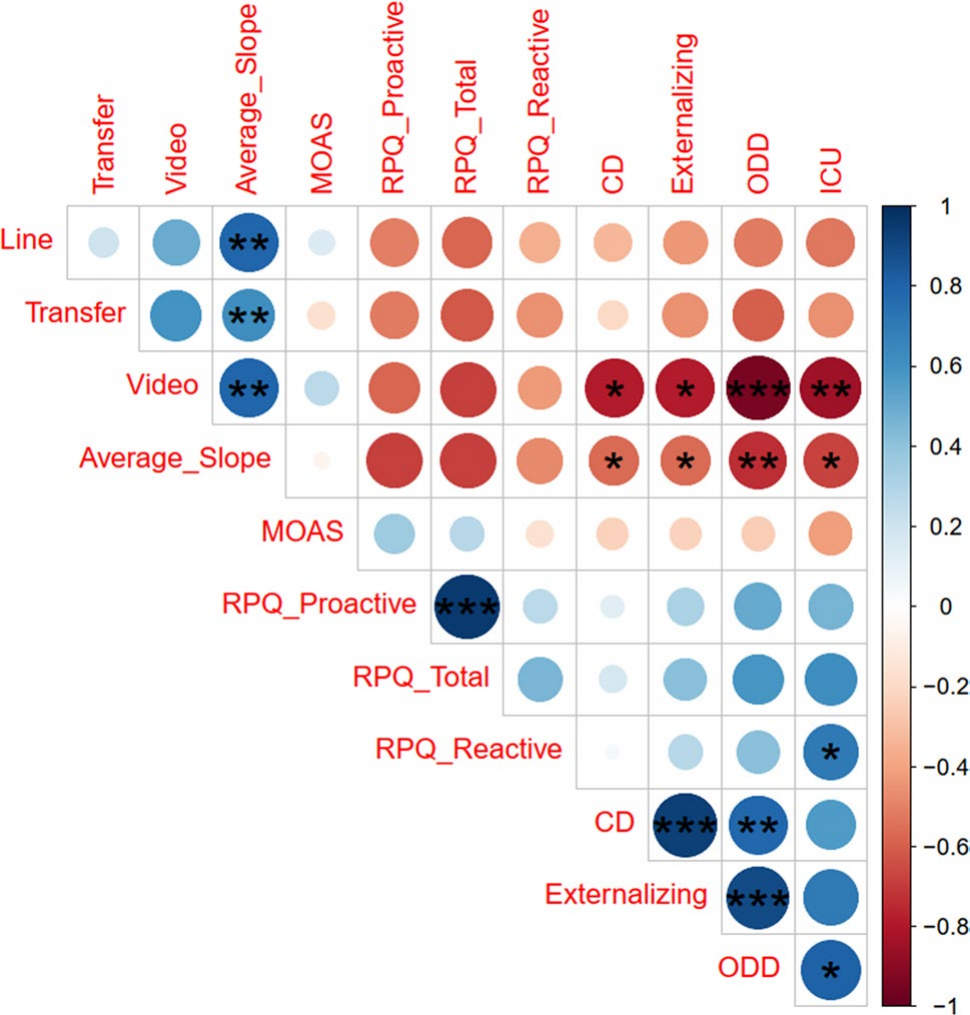
Correlation matrix for SCL Self-regulation and clinical outcome. Correlation Matrix for SCL Self-regulation (slope of the SCL-BF sessions over time) and clinical improvement. Line: Slope of line feedback; Video: Slope of video feedback, Average slope = Mean slope of all runs. Questionnaire data based on Post–Pre differences. RPQ: Reactive–proactive questionnaire; CD: CBCL conduct disorder scale; ODD: CBCL oppositional defiant disorder scale; Externalizing: CBCL Externalizing scale; ICU: Inventory of callous-unemotional traits

As expected, the learning of self-regulation during the video condition and for the mean across all conditions was related to clinical improvement. Lower externalizing symptoms (mean self-regulation: *r*_*s*_ = − 0.621, *p* = 0.041, video condition: *r*_*s*_ = − 0.726, *p* = 0.011), ODD (mean self-regulation: *r*_*s*_ = − 0.761, *p* = 0.007, video condition: *r*_*s*_ = − 0.852, *p* = 0.001), ICU (mean self-regulation: *r*_*s*_ = − 0.621, *p* = 0.041, video condition: *r*_*s*_ = − 0.697, *p* = 0.017), and CD (mean self-regulation: rs = − 0.696, *p* = 0.017, video condition: *r*_*s*_ = − 0.682, *p* = 0.021), but were unrelated to the primary outcome. For details, Fig. 4. We additionally correlated clinical outcome with the number of attended sessions for the completers, which however, were not significant (all *p* > 0.160) Fig. 5.

## Discussion

This first randomized-controlled clinical trial of an individualized SCL-BF training indicated no differences for the primary aggression outcome compared to an active control treatment (TAU) in children and adolescents with DBD. While significant within-group improvements in clinical aggression (both on the primary and other secondary outcomes) were found in both groups, medium-to-large effects were only found in the BF group with respect to the secondary outcomes. Furthermore, this improvement was linked to increased self-regulation in the BF group.

Previous work showed medium-to-large effects of psychosocial behavioral and parent interventions for aggression [4, 23]. However, the control arm in these studies had mostly been a passive/non-active control condition, being limited to waiting list groups that do not receive any treatment. This is the first RCT providing evidence for BF not being an inferior treatment option when compared to a more active control group. Furthermore, the exploratory within-group analysis indicated even higher effect sizes in all secondary outcomes measures in the BF group suggesting that BF might be an alternative treatment option particularly for those children and adolescents with lower IQ who may profit less from cognitive–behavioral treatment. One additional advantage of BF could be the possibility of carrying it out in different settings, such as home settings [20], during a virtual reality or gaming environment [9], with the latter ones possibly enabling better transfer in daily life activities and higher treatment adherence. These types of alternative treatment options might engage and motivate individuals with poor compliance. Nevertheless, this must be interpreted with caution and warrants replication in a larger sample.

This was also the first study to implement an individualized BF treatment for DBD, which fits in recent developments regarding personalized interventions. Such individualized biofeedback has just recently been implemented for clinical samples and has shown promising, or even superior effects when compared to usual BF [5, 11]. DBD is a very heterogeneous disorder with a broad range of clinical and neurobiological manifestations. For example, given that even contrary neurobiological findings have emerged depending on the aggression subtype with higher arousal-related amygdala activity seen in the reactive subtype and lower amygdala activity in the CU subtype [2, 28], individualized arousal treatments are especially warranted in this patient group and need further investigation. However, on a psychophysiological level, our arousal subtyping approach revealed lower mean SCL as expected and no distinct aggression-related subtype profile [3], thus leading to classify 11 out of 13 participants into the group receiving more up-regulation trials. Since our subtyping cut-off was based on an older study [27], we decided in our study design to train in both directions addressing in a flexible manner possible incorrect cut-off values, which additionally promoted differential self-regulation ability of the SCL. Nevertheless, this finding emphasizes the need for further studies.

Regarding the self-regulation of peripheral measures, such as SCL, we showed that participants were able to volitionally self-regulate their SCL. These results are in line with the few trials which reported successful SC biofeedback in psychiatric [24, 25] and neurological [17] disorders. In our study, at a group level, self-regulation significantly improved for the up-regulation condition over time. This might not be surprising, since the majority of participants trained more up-regulation. In addition, improvement of self-regulation skills was related to some secondary clinical outcomes, which adds evidence for specificity. Interestingly, our training condition, which included a more realistic environment through affective video sequences, showed the highest correlations with clinical improvements. In line with this, future studies should explore aggression-related treatments in more ecologically valid contexts, such as home-settings, virtual reality or gaming elements. Furthermore, participants showed significant improvement in self-regulation after the 10th session, which might indicate a minimum amount of ten sessions required for successful improvement on a behavioral level.

## Limitations

As a limitation, we first have to consider that our sample size was small, therefore limiting the robustness of our results. In addition, our TAU group received a relatively low number of treatment sessions, but effect sizes were still in line with more intensive treatments including more sessions. It is important to consider that participants in both groups showed limited compliance, which is typically seen in this patient group, and needed intense contact with the study members, even to complete at least the minimum of six treatment sessions in TAU. One might argue that in the SCL–BF group, the contact with the study members was more intense, since these participants attended more (at least 10) sessions and therefore might have profited from an additional nonspecific effect of assistance and support on treatment outcome. Additionally, it should be pointed out that 20 training sessions with a highly uncompliant population, such as ODD/CD youth, were difficult to carry out, and in some sessions, participants had temper tantrums and tried to quit sessions before completing the whole training. This might also have impacted SCL self-regulation performance during the training sessions. Furthermore, parents or caregivers were not blinded, neither to the SCL-BF nor to TAU, which may well have impacted our results.

## Conclusion

Taken together, our findings showed that individualized SCL-BF was at least as effective as treatment as usual on most treatment outcomes, with nominal but non-significant advantages over TAU in all aggression-related outcomes. Furthermore, BF showed the largest effects on clinical aggression, which depended on the ability to learn to self-regulate the SCL, indicating specificity for arousal-related aggression. This small RCT thus showed promising specific results of a personalized arousal SCL-BF treatment warranting further studies with larger samples and improved methods, for example, by developing BF for mobile use in more ecologically valid settings like at home and in school using wearables.

## Supplementary Information

Below is the link to the electronic supplementary material.

Supplementary file1 (DOCX 133 KB)

Supplementary file2 (DOCX 207 KB)

## Data Availability

The data that support the findings of this study are available from the corresponding author upon reasonable request.
