# Supplementary material for: Randomized controlled trial of individualized arousal-biofeedback for children and adolescents with disruptive behavior disorders (DBD)
Source: Eur Child Adolesc Psychiatry. 2024 Feb 8;33(9):3055–66. doi: 10.1007/s00787-023-02368-5 (PMC11424738; doi:10.1007/s00787-023-02368-5)
Supplement: Supplementary file 2 — Supplementary file2 (DOCX 207 KB) [file 787_2023_2368_MOESM2_ESM.docx]

Supplementary Material

Randomized Controlled Trial of Individualized Arousal-Biofeedback for Children and Adolescents with Disruptive Behavior Disorders (DBD)

Pascal-M Aggensteiner^1^, Boris Böttinger^1^, Sarah Baumeister^1^, Sarah Hohmann^1^, Stefan Heintz^1^, Anna Kaiser^1^, Alexander Häge^1^, Julia Werhahn^2,3^, Christoph Hofstetter^2,3^, Susanne Walitza^2,3^, Barbara Franke^4,5^, Jan Buitelaar^4,5^, Tobias Banaschewski^1^, Daniel Brandeis^1,2,3*^ & Nathalie E Holz*^1,4,5,6^

MOAS scoring: The MOAS, like the OAS (Yudofsky et al., 1986) on which it is based, is designed to measure four types of aggression (verbal, against objects, against self, against others) by severity and frequency, with each type having a rating of zero when the type of aggression was absent, and four levels of severity adding a measure of frequency (f) to each behavior that occurred during the observation period, being 0 = absent, 1 = less than 10 incidents, and 2 = 10 or more incidents. Further, each level of severity had a different weight, being A=0, B=2, C=4 and D=8. The overall aggression score (V) was calculated as V = fA+fB+fC+fD. The score can range between 0 and 40.

Randomization, blinding and harms:
The participants were assigned to groups in a randomized manner, adhering to a 1:1 ratio. Allocation of participants to each group was conducted by an independent and blinded individual who had no direct involvement in the study. Participants and parents were not blinded, because they were instructed according to their group assignment. Throughout the study, no adverse effects were observed.


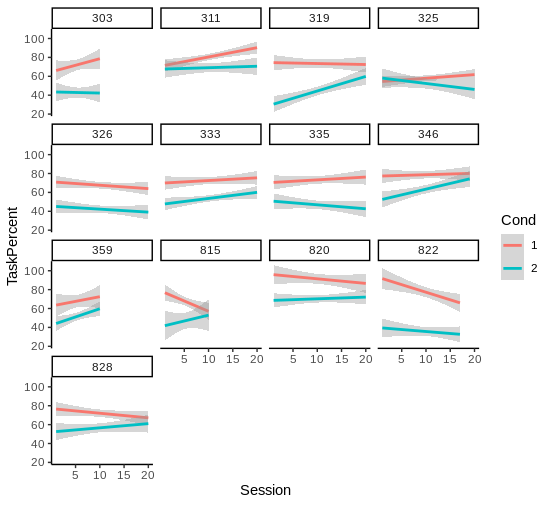
Figure S1 - SCL-BF Individual performance

Mean performance (% correct trials) for each participant.


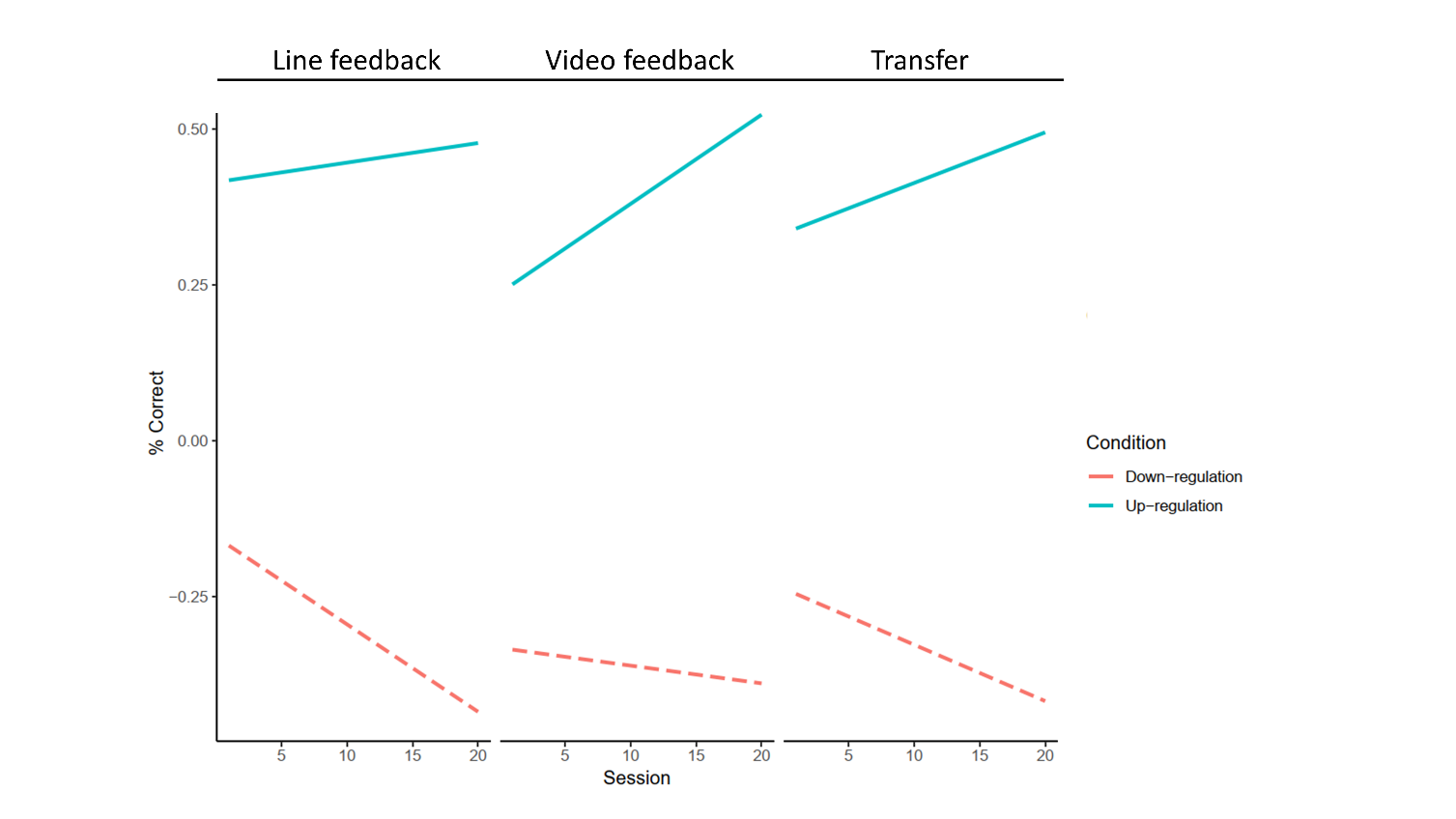


Figure S2 - SCL-BF Performance (offline preprocessed SCL Data)


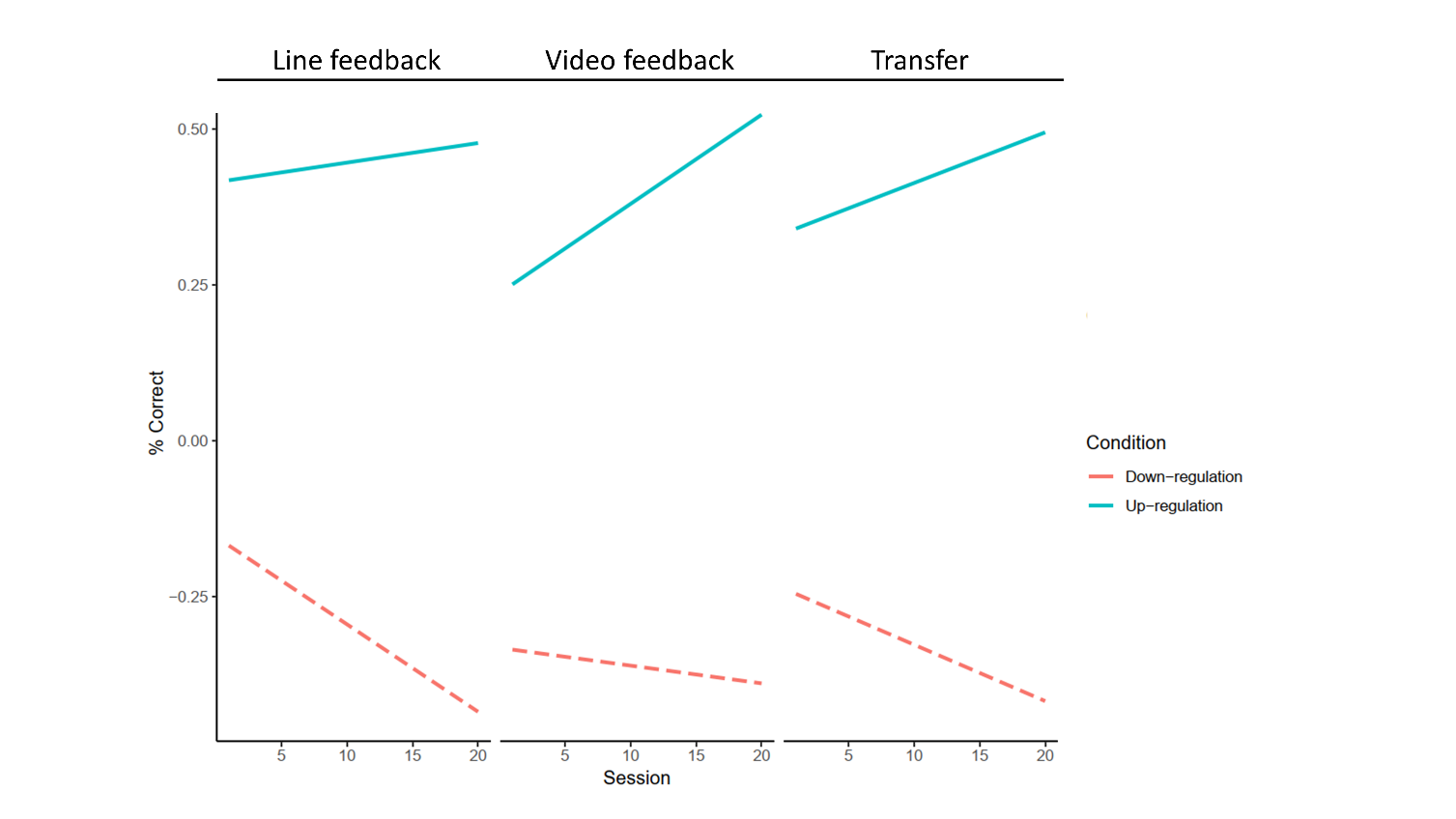


µS

Mean performance (offline preprocessed SCL Data). Each trial consisted of a baseline (10 sec), which was the threshold to up or down - regulate SCL. Each trial lasted 40 sec. µS reflects the mean of down-regulation for each trial minus baseline

Table S1 – Mixed model with offline preprocessed SCL Data (micro Siemens)

|  | | **Task (µ Siemens)** | | |
| --- | --- | --- | --- | --- |
| *Predictors* | *Estimates* | | *CI* | *p* |
| (Intercept) | -0.15 | | -0.38 – -0.03 | 0.128 |
| Session | -0.01 | | -0.02 – 0.00 | 0.123 |
| Run [2] vs Run [1] | -0.18 | | -0.19 – 0.05 | 0.139 |
| Run [3] vs Run [1] | -0.08 | | -0.15 – 0.09 | 0.495 |
| Cond [Up] vs Cond [Down] | 0.57 | | 0.37 – 0.76 | **<0.001** |
| Session * Cond [Up] | 0.02 | | -0.00 – 0.03 | **0.051** |
| Session * Run [2] | 0.01 | | -0.01 – 0.03 | 0.298 |
| Session * Run [3] | 0.00 | | -0.02- 0.03 | 0.644 |
|  |  | |  |  |
|  |  | |  |  |
|  |  | |  |  |

Full model using the online preprocessed SCL Data.

Table S2 – Mixed model taking into account medication (accuracy rates)

|  | | **Task Percent** | | |
| --- | --- | --- | --- | --- |
| *Predictors* | *Estimates* | | *CI* | *p* |
| (Intercept) | 79.19 | | 72.60 – 85.79 | **<0.001** |
| Medication | -4.34 | | -15.69 – 7.01 | 0.454 |
| Session | -0.26 | | -1.04 – 0.51 | 0.503 |
| Run2 [2] | -0.07 | | -3.89 – 3.75 | 0.972 |
| Run2 [3] | -4.57 | | -11.24 – 2.10 | 0.179 |
| Cond2 [2] | -23.70 | | -27.23 – -20.18 | **<0.001** |
| Session * Cond2 [2] | 0.32 | | 0.01 – 0.64 | **0.046** |
| Session * Run | 0.11 | | -0.20 – 0.42 | 0.474 |
| Medication * Session | -0.10 | | -1.07 – 0.88 | 0.845 |
| Medication * Run | -1.74 | | -6.12 – 2.64 | 0.436 |
| Medication * Session *Run | 0.05 | | -0.34 – 0.45 | 0.792 |

Full model including medication as a covariate

SCL- BF Instructions


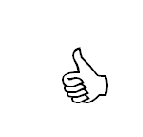
Your task is to figure out how to move the thermometer up (arrowhead pointing upward) or down (arrowhead pointing downward). Some children do this by imagining pictures/emotions or thinking about something specific (e.g., exciting situations or calming/boring feelings). Now, you must discover through practice what works best for you.

Don't be disappointed if it doesn't work right away!

If you practice well, you will also receive a reward! It's important that you stay calm in your chair and don't slide around or fidget.


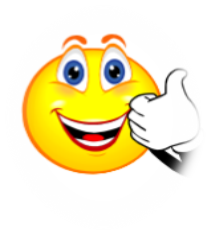


Pre – Post – Follow-Up Data

| *MOAS* | | | | |
| --- | --- | --- | --- | --- |
| Group | | N | Mean | SD |
| SCL-BF | Pre | 17 | 10,41 | 8,783 |
|  | Post | 14 | 5,64 | 6,500 |
|  | FU | 6 | 5,33 | 5,39135 |
| TAU | Pre | 18 | 8,39 | 7,180 |
|  | Post | 12 | 5,00 | 5,063 |
|  | FU | 6 | 5,16 | 9,28260 |

| *CBCL – Parent ODD Total score* | | | | |
| --- | --- | --- | --- | --- |
| Group | | N | Mean | SD |
| SCL-BF | Pre | 18 | 7,28 | 1,841 |
|  | Post | 12 | 5,08 | 2,466 |
|  | FU | 4 | 4,75 | 3,20156 |
| TAU | Pre | 18 | 6,72 | 1,708 |
|  | Post | 12 | 5,50 | 2,236 |
|  | FU | 5 | 6,20 | 2,58844 |

| *CBCL – Parent CD Total score* | | | | |
| --- | --- | --- | --- | --- |
| Group | | N | Mean | SD |
| SCL-BF | Pre | 18 | 12,67 | 6,544 |
|  | Post | 12 | 5,67 | 5,348 |
|  | FU | 4 | 4,50 | 3,69685 |
| TAU | Pre | 18 | 11,11 | 4,825 |
|  | Post | 12 | 9,08 | 4,776 |
|  | FU | 5 | 8,80 | 8,22800 |

| *ICU – Parent Total score* | | | | |
| --- | --- | --- | --- | --- |
| Group | | N | Mean | SD |
| SCL-BF | Pre | 17 | 33,24 | 9,852 |
|  | Post | 12 | 28,50 | 11,981 |
|  | FU | 5 | 31,80 | 9,03881 |
| TAU | Pre | 18 | 33,11 | 7,388 |
|  | Post | 12 | 33,58 | 7,229 |
|  | FU | 5 | 37,00 | 8,77496 |
